# Long-term Survival After Treating Cardiac Metastasis With Radiation and Immune Therapy: A Case Report

**DOI:** 10.7759/cureus.2607

**Published:** 2018-05-10

**Authors:** W. Tristram Arscott, Priti Lal, Ronac Mamtani, Rupal O'Quinn, Rajat Deo, Joshua Jones

**Affiliations:** 1 Radiation Oncology, Hospital of the University of Pennsylvania; 2 Pathology, Hospital of the University of Pennsylvania; 3 Oncology, Hospital of the University of Pennsylvania; 4 Cardiology, Hospital of the University of Pennsylvania; 5 Radiation Oncology, University of Pennsylvania

**Keywords:** urothelial carcinoma, cardiac metastasis, cardiac radiation, immune therapy

## Abstract

Cardiac metastases are a rare clinical entity and they generally portend a poor prognosis. Management is generally directed toward symptom control and maintaining cardiac function; however, long-term survival is rare. Here, we report a case of isolated metastatic urothelial cell carcinoma to the right ventricle that was functionally limiting the patient. The metastasis was successfully palliated for 17 months following radiation and immune therapy; however, disease progression in and around his heart ultimately led to a cardiac arrest.

## Introduction

Cardiac metastases are a rare clinical entity and they generally portend a poor prognosis. Patients often present symptomatic from cardiac involvement, either by functional impairment of blood flow from the mass itself (syncope, heart failure, emboli), or arrhythmias/conduction delays, or pericardial effusions. Cardiac metastases almost always indicate systemic disease and are seldom a solitary event [1-2]. Management is generally directed toward symptom control and maintaining cardiac function; however, long-term survival is rare, with a median survival around 3.5 months without treatment [1,3-4]. Here, we report a case of isolated metastatic urothelial cell carcinoma to the right ventricle. The patient was treated with conformal radiotherapy followed by program death-ligand 1 (PD-L1) targeted therapy (atezolizumab). He developed significant treatment-related toxicity; however, he was able to recover after multidisciplinary symptom management. He demonstrated an exceptional and durable response for 17 months; however, disease progression in and around his heart ultimately led to a cardiac arrest.

## Case presentation

**Figure 1:**
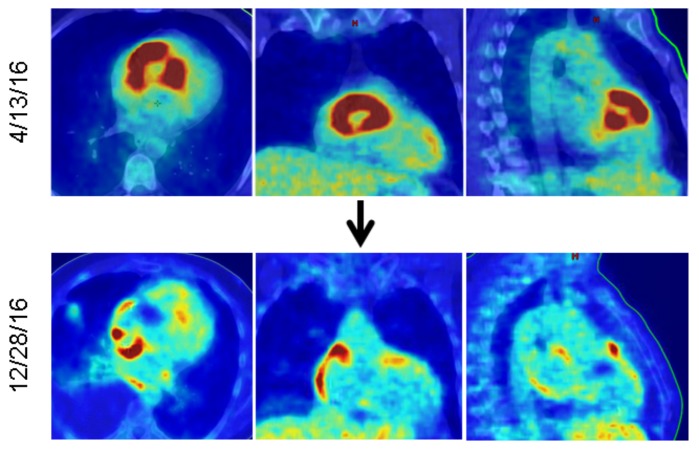
PET/CT fusion scan showing the FDG-avid mass (red) in the right ventricle The upper panel shows the appearance of the mass at the time of recurrence, the lower panel is seven months after completing radiotherapy. Views from left to right are: axial, coronal, and sagittal. PET: positron emission tomography; CT: computed tomography; FDG: fluorodeoxyglucose.

A 51-year-old male was diagnosed with muscle-invasive urothelial cell carcinoma in May 2015. Standard neoadjuvant chemotherapy was given followed by radical cystoprostatectomy. Final pathology showed pT3 N0 M0, stage III disease. He remained disease free until April 2016, at which point he developed new exertional dyspnea and a small lung nodule was noted on imaging. During cardiac clearance for a biopsy, a Mobitz 2 heart block with bradycardia was noted. Transthoracic echocardiogram (ECG) demonstrated a mass in the right ventricular outflow tract, which was additionally found to be fluorodeoxyglucose (FDG)-avid on positron emission tomography (PET) computed tomography (CT) (Figure 1, upper panel).

**Figure 2:**
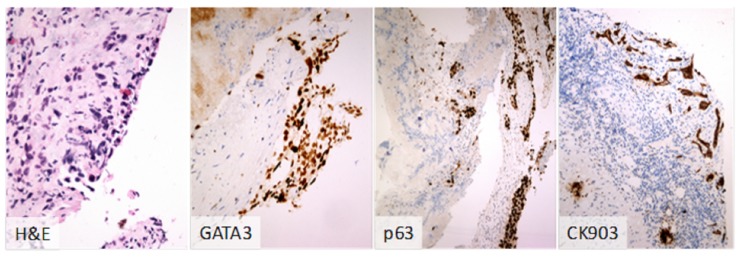
Cardiac mass biopsy The cardiac biopsy revealed multiple clusters of atypical cells which were positive for GATA 3, P63, and CK903. The patient’s history of urothelial carcinoma along with the immunomorphologic findings were consistent with metastatic urothelial carcinoma to the heart.

The patient developed progressive dyspnea with minimal exertion and then experienced a syncopal episode prompting hospitalization. Biopsy of the right ventricular mass demonstrated poorly differentiated carcinoma, consistent with urothelial origin (Figure 2). 

**Figure 3:**
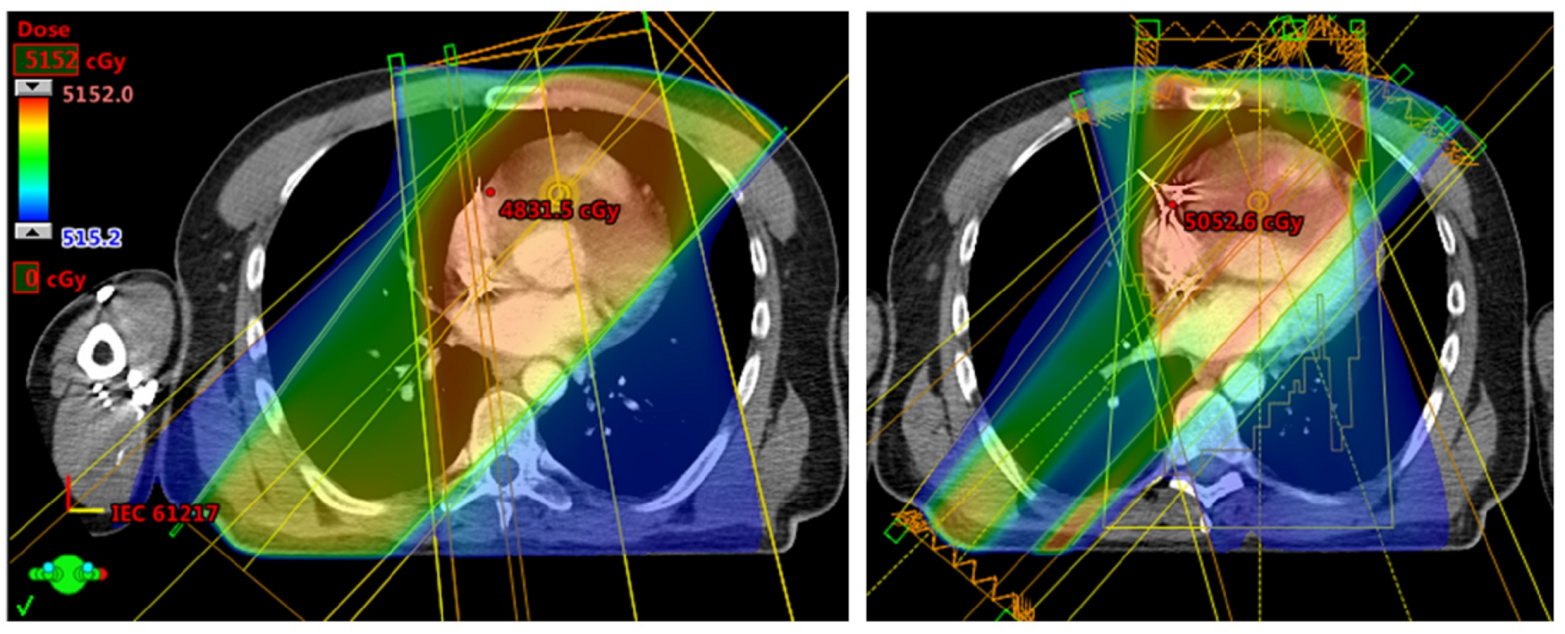
Radiotherapy planning 3D conformal radiotherapy plan (3DCRT) to the left and intensity modulated radiotherapy plan (IMRT) on the right, demonstrating how advanced planning was used to reduce dose to the uninvolved left side of the heart.

The mass was not felt to be respectable and chemotherapy was not felt to offer rapid disease control in the setting of progressive symptoms. He was offered palliative radiotherapy. His bradycardia progressed to a complete heart block, likely due to the growth of the mass, necessitating placement of a dual chamber pacemaker. The right ventricle mass was treated with 45 Gy in 18 fractions (3D conformal photons for 5 fractions, followed by intensity-modulated radiotherapy for the remaining 13 fractions (to reduce dose to the left ventricle). Figure 3 demonstrates the dose distribution.

Proton radiation was considered to spare the uninvolved myocardium. He had substantial tumor thromboembolic disease to his lungs during radiotherapy, requiring medical intensive care unit (MICU) admission for symptom management. He ultimately completed the full course of radiotherapy which improved his functional status.

Following radiotherapy, he began immune checkpoint therapy with the PD-L1 antagonist atezolizumab (1200 mg every three weeks), which was well tolerated. A PET/CT scan seven months after completing radiation demonstrated a complete metabolic response in the right ventricle mass and no other sites of progression (Figure 1, lower panel). The previously noted pulmonary nodules were stable. He resumed normal activity and returned to work. Eight months after radiotherapy and six months into his immune therapy, he developed progressive shortness of breath. He had heart failure and the ejection fraction reduced to 20%-30%. Cardiac magnetic resonance imaging (MRI) revealed a focal area of subendocardial delayed enhancement, potentially representing myocarditis. Cardiac catheterization revealed extensive coronary artery disease and complete occlusion of the right coronary artery; however, the left to right collaterals were intact. Atezolizumab was held with an initiation of high dose steroids to treat potential immune-mediated myocarditis with minimal effect. Electrophysiology testing demonstrated severe cardiac dyssynchrony. Cardiac resynchronization therapy was recommended and a biventricular pacer was placed with rapid symptom relief. He returned to work with overall minimal symptoms. Atezolizumab was resumed, and a total of 18 cycles (54 weeks) of therapy were completed without issue.

**Figure 4:**
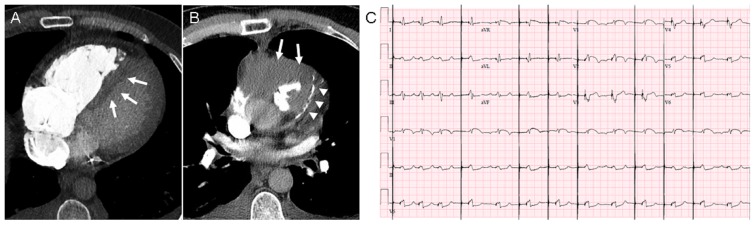
Progressive disease in and around the heart with ECG changes Computed tomography (CT) scan with contrast timed for pulmonary embolism evaluation. (A) Contrast material filling the right ventricle with bowing of the interventricular septum (arrows) towards the left ventricle, consistent with elevated right heart pressure. (B) Tumor progression around the heart (arrows) and surrounding the LAD (arrowheads). (C) ECG showing ST elevations in the anterior leads (V1-V3) correlating with known disease progression involving the LAD. LAD: left anterior descending; ECG: echocardiogram.

In the months that followed the placement of his biventricular pacer, he was evaluated several times for shortness of breath and atypical chest pain, however with no clear etiology. PET/CT imaging in August 2017 demonstrated increased FDG-avidity in his right ventricle concerning for progression. An MRI was ordered and in early September 2017, he was admitted for workup of an exacerbation of chest pain. A contrast-enhanced CT of the chest showed significant disease progression in the area of prior treatment, extension toward the left ventricle and along the heart wall leading to increased right heart pressure (Figure 4A), and probable encasement of his left anterior descending (LAD) artery by disease progression (Figure 4B). He had acute progression of chest pain associated with an increased oxygen requirement, shortly after being admitted. His ECG showing ST elevations in the anterior leads, consistent with an LAD infarct (Figure 4C). The cardiology service was consulted to consider palliative LAD stenting; however, the patient underwent cardiac arrest in the interim and did not wish to be resuscitated. He died 17 months after his disease recurrence.

## Discussion

The prognosis of patients with cardiac metastases is uniformly poor, with few long-term survivors. A cardiac metastasis as a solitary site of recurrence is particularly uncommon [1]. A recent autopsy series estimated the incidence of autopsy-identified cardiac metastases in cancer patients to be 7.1% (vs. 2.1% in the general population) [5], and the incidence is anticipated to rise owing to improved survival with modern therapies. Among patients with urothelial cancer, cisplatin-based chemotherapy shows an initial favorable response in the metastatic setting; however, few responses are durable [6]. Recent trials of programmed death receptor-1 (PD-1)/PD-L1 inhibitors have demonstrated durable responses with long-term survival, and five immune checkpoint inhibitors have received FDA approval in this disease, including atezolizumab [7-10]. Radiation has rarely been used with success in the treatment of cardiac metastases, and only a limited number of successfully treated cases and techniques have been documented [2,4-5]. Additionally, radiotherapy increases the presentation of antigens to immune cells, potentially enhancing T-cell killing of tumor cells [11]. Toxicity from cardiac radiation and immune therapy combinations have not been reported.

This case is notable for several reasons. First, the patient was exquisitely symptomatic and functionally limited due to the metastasis in the right ventricle when it was first identified (dyspnea on exertion followed by a syncopal episode). This was presumably due to the right ventricle outflow tract obstruction by the mass during exertional activity (a ball-valve type of obstruction), though this may have been influenced by his pulmonary tumor emboli as well. Second, the patient presented within a year of his initial diagnosis, and was a poor surgical candidate, making the optimal management of his disease a challenge. Third, his long-term survival is noteworthy, a sign of both effective upfront management of his metastatic disease and that durable responses can be achieved with immune therapy. Lastly, the side effects from the treatment of his metastatic disease (both during and after radiation) were profound and debilitating. The risk of tumor emboli during the treatment of cardiac metastases was discussed [2], with few options for management other than careful observation and management of symptoms if they develop; this was apparent in our patient and nearly caused his death during radiotherapy. The heart failure that developed later required thoughtful multidisciplinary discussions and interventions, ultimately enabling him to resume normal daily activity as well as continue his work up until the month of his cardiac arrest.

His recurrent atypical chest pain was evaluated numerous times but without a clear etiology. It was thought, maybe, to reflect his tumor burden/treatment scar versus recurrent pericarditis (which he experienced during radiotherapy). While the PET/CT had shown an initial favorable response, the high metabolic activity of the myocardium potentially clouded the evaluation of the extent of response to the treatment. At least in hindsight, earlier evaluation with other imaging modalities (contrast-enhanced CT or cardiac MRI) may have identified the extension of the mass towards the left ventricle and around the heart, allowing for prophylactic stenting of the left anterior descending artery. Whether this would have truly prolonged his life given his disease progression is not known.

## Conclusions

Patients with cardiac metastases seldom demonstrate long-term survival. Here, we describe a patient with cardiac metastasis managed with radiotherapy and immunotherapy who experienced long-term disease control. Long-term survival even in the metastatic setting is becoming more common, particularly in the expanding use of immunotherapy. Given that immunotherapy presents unique safety profiles that differ from conventional cytotoxic chemotherapy, it may likewise demonstrate distinct toxicities when combined with radiotherapy. Multidisciplinary team involvement when determining the use of these newly combined interventions and for the evaluation of treatment toxicities in follow up is essential. This will play a key role in enabling the broader use of these therapies, including their application in advanced stages of the disease.
